# Detection and genomic analysis of *BRAF* fusions in Juvenile Pilocytic Astrocytoma through the combination and integration of multi-omic data

**DOI:** 10.1186/s12885-022-10359-z

**Published:** 2022-12-12

**Authors:** Melissa Zwaig, Audrey Baguette, Bo Hu, Michael Johnston, Hussein Lakkis, Emily M. Nakada, Damien Faury, Nikoleta Juretic, Benjamin Ellezam, Alexandre G. Weil, Jason Karamchandani, Jacek Majewski, Mathieu Blanchette, Michael D. Taylor, Marco Gallo, Claudia L. Kleinman, Nada Jabado, Jiannis Ragoussis

**Affiliations:** 1 McGill Genome Centre and Department of Human Genetics, McGill University, Montreal, Canada; 2 Quantitative Life Sciences and Lady Davis Institute for Medical Research, Montreal, Quebec Canada; 3 Alberta Children‘s Hospital Research Institute, Charbonneau Cancer Institute, and Department of Biochemistry and Molecular Biology, Cumming School of Medicine, University of Calgary, Calgary, AB Canada; 4 Department of Human Genetics and Lady Davis Institute for Medical Research, Jewish General Hospital, Montreal, Quebec Canada; 5 The Research Institute of the McGill University Health Centre, Montreal, Canada; 6 Department of Pathology, Centre Hospitalier Universitaire Sainte-Justine, Université de Montréal, Montréal, QC, H3T 1C5 Canada; 7 Department of Pediatric Neurosurgery, Centre Hospitalier Universitaire Sainte-Justine, Université de Montréal, Montréal, QC H3T 1C5 Canada; 8 Department of Pathology, Montreal Neurological Institute, McGill University, Montreal, QC H3A 2B4 Canada; 9 School of Computer Science and McGill Center for Bioinformatics, McGill University, Montréal, Québec Canada; 10 Arthur and Sonia Labatt Brain Tumour Research Centre, Hospital for Sick Children Research Institute, Toronto, Canada; 11 Department of Human Genetics, Department of Pediatrics, and The Research Institute of the McGill University Health Centre, Montreal, Canada

**Keywords:** Juvenile Pilocytic Astrocytoma, *BRAF* fusions, Linked-reads, Hi-C, RNA sequencing, Single-cell RNA sequencing

## Abstract

**Background:**

Juvenile Pilocytic Astrocytomas (JPAs) are one of the most common pediatric brain tumors, and they are driven by aberrant activation of the mitogen-activated protein kinase (MAPK) signaling pathway. *RAF*-fusions are the most common genetic alterations identified in JPAs, with the prototypical *KIAA1549-BRAF* fusion leading to loss of *BRAF*’s auto-inhibitory domain and subsequent constitutive kinase activation. JPAs are highly vascular and show pervasive immune infiltration, which can lead to low tumor cell purity in clinical samples. This can result in gene fusions that are difficult to detect with conventional omics approaches including RNA-Seq.

**Methods:**

To this effect, we applied RNA-Seq as well as linked-read whole-genome sequencing and in situ Hi-C as new approaches to detect and characterize low-frequency gene fusions at the genomic, transcriptomic and spatial level.

**Results:**

Integration of these datasets allowed the identification and detailed characterization of two novel *BRAF* fusion partners, *PTPRZ1* and *TOP2B,* in addition to the canonical fusion with partner *KIAA1549*. Additionally, our Hi-C datasets enabled investigations of 3D genome architecture in JPAs which showed a high level of correlation in 3D compartment annotations between JPAs compared to other pediatric tumors, and high similarity to normal adult astrocytes. We detected interactions between *BRAF* and its fusion partners exclusively in tumor samples containing *BRAF* fusions.

**Conclusions:**

We demonstrate the power of integrating multi-omic datasets to identify low frequency fusions and characterize the JPA genome at high resolution. We suggest that linked-reads and Hi-C could be used in clinic for the detection and characterization of JPAs.

**Supplementary Information:**

The online version contains supplementary material available at 10.1186/s12885-022-10359-z.

## Background

Juvenile Pilocytic Astrocytomas (JPAs) are the most common type of pediatric low-grade glioma (pLGG) [1]. They are characterized by abnormal activation of the mitogen-activated protein kinase (MAPK) pathway mediated by various genetic events [2, 3]. Over 60% of JPAs contain a tandem duplication leading to a fusion between *KIAA1549* and *BRAF* that results in the loss of the auto-inhibitory domain of *BRAF* and the constitutive activation of its kinase domain [2, 4]. Similar fusions leading to constitutive activation of both *BRAF* and *RAF1* (a serine/threonine-specific protein kinase, also part of the *RAF* family) with a variety of 5′ partners have been identified over the last few years, steadily increasing the *RAF* fusion partner spectrum in JPAs as well as in melanomas and solid tumors [2, 3, 5–8]. This wide variety of fusion partners has complicated the molecular diagnostic of JPAs in the clinic, since the classical targeted approach (RT-PCR) fails to capture the full spectrum of fusions.

While survival rates for patients with pLGG are high (the 10-year survival rate is over 90% [2]), in rare instances, they can be challenging to differentiate from higher grade gliomas, especially in infants, and in adolescents and young adults. Indeed, in tumours resected from infants, proliferation rates higher than what is typical in JPA (below 5%) can be encountered. Meanwhile, in adolescents and young adults, a new entity (anaplastic pilocytic astrocytoma) has been recently described which shows more aggressive behavior and less favorable prognosis than JPAs. To improve molecular diagnosis and spare patients’ unnecessary chemo- and radiotherapy, it is imperative that we are able to detect the canonical and novel *RAF* fusions with high sensitivity, particularly in cases were high non-tumor content prevents us from achieving correct molecular diagnosis using standard next-generation sequencing or DNA methylation arrays. Additionally, characterizing the spectrum of *RAF* fusion partners in JPAs would help provide better understanding of the underlying molecular mechanisms leading to these oncogenic events.

To date, many studies looking at JPAs have focused on differential methylation and expression patterns across tumor locations [9–14]. These studies were performed using array technologies able to detect structural variants (SVs) caused by tandem duplications, such as the *KIAA1549-BRAF* fusion [4], or large deletions, such as the ones causing the recurrent *FAM131B-BRAF* fusion [8], but are not able to detect fusions created by copy neutral alterations such as chromosomal translocations and inversions. While large consortium studies that use whole-genome sequencing (WGS) and RNA sequencing (RNA-Seq) have had more success in identifying novel somatic events in pLGG [3, 6], both technologies have their limitations when used to characterize the complex genomic rearrangements giving rise to these fusions. Large SVs (typically described as > 30 kb) are difficult to detect with typical short read sequencing [15] and the low sample purity [9, 10, 16, 17] in some JPAs further complicates SV detection by WGS. When performing RNA-Seq, this limitation may be overcome by high expression of the transcript of interest, however, in JPAs overexpression of constitutively active *BRAF* has been shown to cause oncogene-induced senescence [18]. Additionally, the mechanism by which the fusion was created cannot be deciphered by RNA-Seq.

One potential solution is the use of linked-read sequencing, which tags all reads coming from a single piece of high molecular weight DNA with a shared barcode also called a unique molecular ID (UMI) [19]. This library is then be sequenced using short Illumina reads and the UMIs are used to identify reads originating from the same long DNA molecule which improves alignment. Linked-read technology allows not only the detection of much larger structural variants than short read sequencing alone, but also the generation of haplotype blocks that can be leveraged during analysis. Another potential approach is Hi-C, which allows us to assess the level of the interactions between different genomic regions in 3D space as well as chromatin compaction throughout the genome. Regions of low compaction are transcriptionally active and referred to as type A compartments, while type B compartments are highly compacted and transcriptionally repressed. Compartments of the same type have also been shown to interact with each other to a greater extent than those of a different type [20].

To this effect, we applied RNA-Seq to nine JPA samples in combination with 10x Genomics’ linked-reads in six of these samples to assess its performance in the discovery and detailed characterization of low frequency *BRAF* fusions including two with novel fusion partners *PTPRZ1* and *TOP2B*. We also generated in situ Hi-C libraries from primary JPA tumor samples in order to gain more insight into the association between fusion-partner genes and nuclear topology. Investigation of the Hi-C data allowed us to assess whether the compartments are consistent across JPAs, the accessibility of the regions involved in *RAF* fusions as well as if those regions interact in normal cells.

## Methods

### RNA Sequencing

RNA-Seq data was generated for 9 samples. Our pipeline was build based on the recommendations by Sommerkamp et al. [41]. Reads were aligned with STAR (v2.5.3a) [21] to reference GRCh38_gencode_v33_CTAT_lib_Apr062020 using the following parameters:

*--twopassMode Basic --chimSegmentMin 12 --chimOutType WithinBAM.*

*--alignSplicedMateMapLminOverLmate 0.5 --outFilterMultimapNmax 20.*

*--alignSJoverhangMin 8 --alignMatesGapMax 200,000 --alignIntronMax 200,000.*

*--alignSJDBoverhangMin 10 --alignSJstitchMismatchNmax 5–1 5 5 --outSAMmultNmax 20.*

Fusions were called with three different fusion callers; 1) STAR-Fusion (v1.2.0) using the Chimeric.out.junction file from STAR as input [22], 2) Arriba v1.2.0, [23] using the Chimeric.out.sam, aligned BAM and the COSMIC known Fusion List [24] as input, and 3) InFusion (v0.8.1-dev) using a relaxed sets of parameters *(−-allow-intronic --allow-intergenic --allow-non-coding --allow-all-biotypes*) and a more stringent set *(−min-split-reads 3 --min-span-pairs 2 --min-fragments 4*) [25].

### 10x Genomics Linked-reads

10x Genomics whole-genome linked-read libraries were produced for 6 JPAs and corresponding blood samples. Tumor DNA was extracted either with Chemagen 10 mg Tissue Protocol with overnight external lysis (PerkinElmer, Inc., Waltham, Massachusetts, United States, cat# CMG-723) followed by size selection using the BluePippin PacBio 20 kb cassette or using MagAttract HMW DNA kit (Qiagen, Hilden, Germany) as per protocol (Supplemental Table 4). DNA from the blood was extracted using the Qiagen MagAttract HMW DNA kit followed by size selection with the SageHLS HighPass > 300 kb protocol as needed (Supplemental Table 4). DNA yields were measured by Qubit™ dsDNA BR Assay Kit (ThermoFisher Scientific, cat# Q32853) and molecule length was assessed by Femto Pulse (Genomic DNA 165 kb Kit, 3 hours run, Agilent Technologies, Inc., Santa Clara, California, United States, cat# FP-1002-0275). Samples were diluted to 1 ng/uL and re-quantified in triplicate by Qubit™ dsDNA HS Assay Kit (ThermoFisher Scientific, cat# Q32854). Library preparation was done following the Chromium™ Genome Reagent Kits v2 User Guide (10x Genomics, Pleasanton, California, United States). Final libraries concentrations were measure by qPCR (Roche, Basel, Switzerland, KAPA Library Quantification Kits - Complete kit (Universal), cat# 07960140001) and profiles were assessed using the Caliper LabChip DNA High Sensitivity assay (PerkinElmer, Inc., Waltham, Massachusetts, United States). Each library was sequenced using 150PE Illumina reads on a single lane of the HiSeqX. Average molecule length (as calculated by 10x Genomics’ analysis pipeline) ranged from 19 kb–85 kb for tumor samples and 42 kb–104 kb for blood samples (Supplementary Table 4).

Alignment and variant calling was done using 10x Genomics’ pipeline (LongRanger), which uses the barcode-aware aligner, Lariat. SVs were also called with three other callers designed for linked-reads, GROC-SV [26], NAIBR [27] and LinkedSV [28] as well as SvABA (designed for standard WGS) [29] (Supplementary Table 3). LongRanger, GROC-SV, and NAIBR were run using the exclusion list (blacklist) provided by 10x Genomics. All callers were run using default parameters with specifications for tumor samples used were applicable. Copy number changes were called using the TitanCNA 10x workflow [30], the ploidy was selected manually and plotting for the circos plots was done using the .titan.txt file. All SV and CNA calling was done using the Lariat aligned BAM files generated by the LongRanger pipeline. A custom R script was used to look for SVs detected by more than one callers. SVs calls were deemed to be the same if both the start and end breakpoints fell within ±1000 bp of each other. All SV calls large than 10 kb and detected by at least 2 callers were manually validated using Loupe.

### In situ Hi-C

In situ Hi-C libraries were generated from 5 million dissociated and fixed cells from JPAs primary tumor samples as described in Rao et al. with minor modifications [31]. Briefly, cells were cross-linked with formaldehyde before the DNA was digested using a 4-cutter restriction enzyme (e.g., DpnII) within intact permeabilized nuclei. Next, sticky ends were repaired to blunt ends using biotinylated nucleotides, and ligated together. The DNA was then sheared and the biotinylated ligation junctions were pulled down with streptavidin beads. Library preparation was done using these fragments and sequenced using paired-end Illumina reads. As quality control (QC) steps, we checked for efficient sonication with an agarose DNA gel and for appropriate size selection using the Agilent Bioanalyzer on final amplified libraries. For the final QC, we performed low-depth sequencing on the Illumina HiSeq 2500 (~ 40 M reads/sample) to assess quality of the libraries using percent of reads passing filter, percent of chimeric reads, and percent of forward-reverse pairs before doing further sequencing on the NoveSeq (Supplemental Table 5).

Hi-C files for NPCs, glial cells and neurons were downloaded from Rajarajan et al. [32] (registration required; Data Download—Study “iPSC-HiC”). Raw data for the spinal and cerebellum astrocytes was retrieved from ENCODE [33, 34]. Analysis of Hi-C generated and downloaded fastq files was performed using Juicer and the associated Juicer Tools [35]. Contact maps were generated using Juicer with the following parameters: -s DpnII -g hg38. Map resolution was determined by using Juicer’s “calculate_map_resolution.sh” script. .hic files were converted to cooler files for further processing using hic2cool [36, 37]. Karyotypic aberration-aware normalization factors were then computed using OneD [38] and applied to cooler files as balancing weights. Subsequently, compartment scores were calculated through the eigs-cis module of cooltools [39].

### Generation of 4C interaction profiles from Hi-C matrices

Virtual 4C plots were generated using the sum of interactions with an anchor across the chromosome and were processed through a custom normalization. First, contacts were binned at a resolution of 10 kb. This resolution, however, was too noisy for accurate virtual 4C reconstruction so the bins were then summed to get 50 kb resolution starting at the *BRAF* breakpoint such that the C-terminal that is kept and the N-terminal that is lost were separated into different bins. The two anchors considered were the *BRAF* C-terminal region (chr7: 140710000–140,790,000) and the *BRAF* N-terminal region (chr7: 140790000–140,930,000). A third anchor that spawned the complete *BRAF* region (chr7: 140710000–140,930,000) was used as “background” to normalize the virtual 4C. Thus, the interaction that each 50 kb bin made with either of the two *BRAF* terminals was divided by the total interaction with the full *BRAF* gene, to account for the distance bias between a bin and the anchor.

### Single-cell analysis of mouse cerebellum atlas

We used the developing mouse cerebellum atlas [40] to inspect the expression of *BRAF* and its fusion partner genes throughout development and specifically at embryonic days 16 and 18 when tumor initiation is thought to occur in JPAs (Supplemental Fig. 4). We looked at the average expression and the percentage of cells expressing *BRAF* in all cell types present at embryonic day 16 and 18 as shown in the barplots (Supplemental Fig. 4b-c). The gene expression Dot plot was generated using Seurat’s DotPlot() function [9].

## Results

A total of 14 samples from 13 patients were used in this study (Supplemental Table 1). We generated RNA-Seq data for nine patients and 10x Genomics linked-reads for 6 of these samples. We also generated in situ Hi-C for 10 patients from 11 JPAs. Two samples processed with the linked-reads also had fixed cells available for Hi-C. For three samples, we were able to generate both RNA-Seq and Hi-C, and 5 samples had only fixed cells for Hi-C data available.

### Identification of *BRAF* fusions using RNA-Seq

**Fig. 1:**
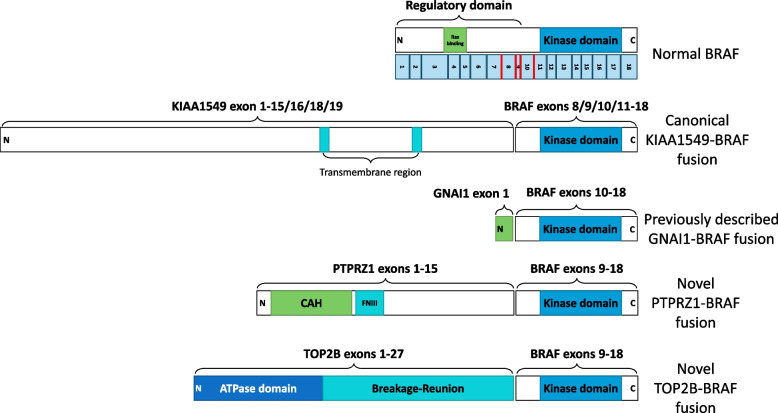
Diagrams representing normal *BRAF* protein and fusions characterized in this study.

We generated RNA-Seq for 9 patients and analyzed them using a custom pipeline, in part based on the recommendations by Sommerkamp et al. [41]. We detected a *BRAF* fusion in all samples, of which 6 contained the *KIAA1549-BRAF* fusion, 1 contained a previously described *GNAI1-BRAF* fusion [3], and two contained fusions with novel partners *PTPRZ1* and *TOP2B* (Fig. 1, Supplemental Table 1). However, in 5 out of the 9 samples, the *BRAF* fusion was detected by only 1 of the three fusion callers used out of a total of 429–1296 calls (Supplemental Table 2, Additional file 2).

### Identification of *BRAF* fusions and their underlying genomic mechanisms using linked-reads

**Fig. 2:**
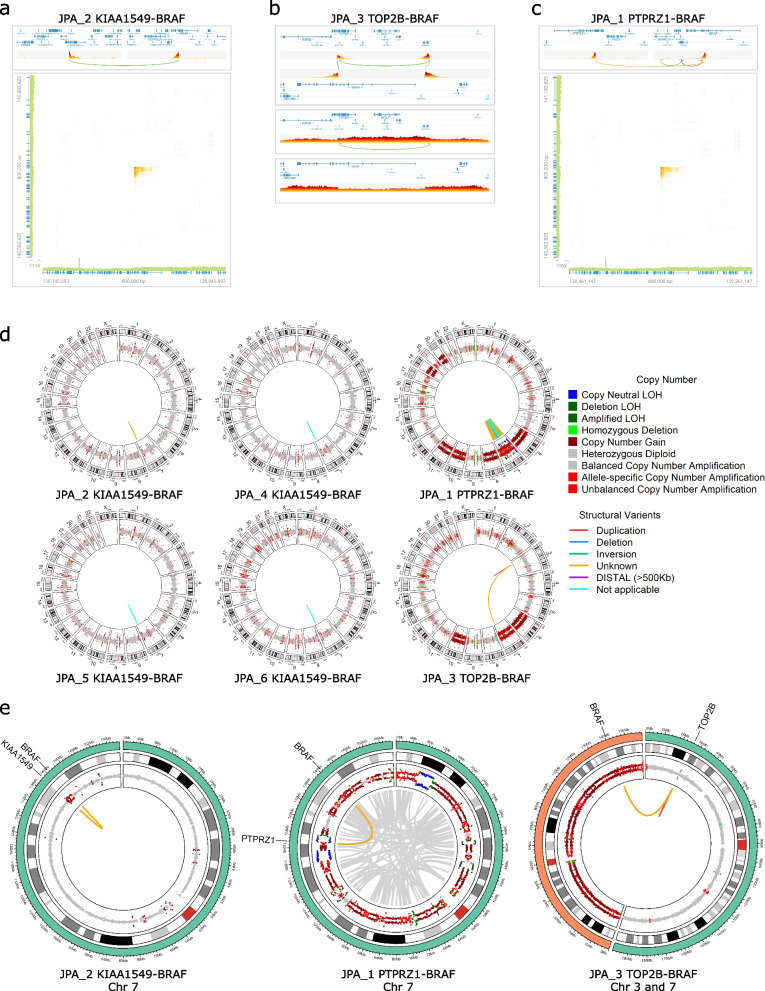
Linked-reads data supporting *BRAF* fusions created by (**a**) a tandem duplication resulting in the canonical *KIAA1549-BRAF* fusion in JPA_2, (**b**) an interchromosomal translocation creating a *TOP2B-BRAF* fusion detected in JPA_3, and (**c**) a structural variant leading to the *PTPRZ1-BRAF* fusion detected in JPA_1, all visualized in Loupe. **d** Circos plots for linked-read datasets showing CNV calls made by TitanCNA (outer circle) and manually validated somatic SVs detected by at least two linked-read callers (inner circle). Copy number abnormalities are colour coded; structural variants are indicated by lines. **e** Circos plot of chromosome 7 in JPA_2 showing the canonical fusion, a catastrophic chromosomal event on chromosome 7 in JPA_1 and circos plot of chromosomes 3 and 7 with an interchromosomal fusion in JPA_3. Circos plots for linked-read datasets showing CNV calls made by TitanCNA (outer circle) and manually validated somatic SVs detected by at least two callers (inner circle). Copy number abnormalities are colour coded; *BRAF* fusions are shown in yellow and all other SVs in grey

Tumor material was available for 6 out of 9 tumors that were profiled by RNA-Seq (Supplemental Table 1). We generated 10x Genomics linked-read data and developed a multi-caller analysis approach, which allowed us to generate a set of high confidence SVs that could then be manually validated as somatic (see Methods, Supplemental Table 3). Using this pipeline, we detected SVs in all samples which supported the fusions we identified by RNA-Seq, including 4 cases of the canonical fusion and both novel fusions, by at least 2 callers (Fig. 2a-c, Supplemental Fig. 1, Supplemental Table 1, Additional files 3-8).

The canonical fusion between *KIAA1549* and *BRAF*, caused by a tandem duplication at 7q34, was first described in 2008 using a combination of bespoke microarray technologies and iFISH [4]. It has since been found in 60–70% of JPAs including 66% of samples in this study (Fig. 2a, Supplemental Fig. 1). Each tandem duplication was supported by 80–100% of barcodes on the assigned haplotype that support the SV (HAP_ALLELIC_FRAC as calculated by LongRanger).

While the novel *TOP2B-BRAF* fusion was first detected by RNA-Seq, further investigation with linked-read data showed a second breakpoint upstream of the first and a duplication on chromosome 3 between the two events (Fig. 2b). The SVs were supported by 95.6 and 96.0% of barcodes on the assigned haplotype (as calculated by LongRanger) for the *BRAF* fusion causing SV and downstream SV, respectively. Using the phasing information provided by the linked-reads, we found that both breakpoints occurred on the same haplotype of chromosome 7, indicating a single event. We hypothesized that a 151-kb segment of chromosome 7, containing the 5′ end of *BRAF*, was lost and a 263-kb segment of chromosome 3, was inserted in its place using the homology directed repair pathway. A 143 kb deletion on 7q34 (including *BRAF*) was detected by array CGH at diagnosis, however, the translocation and subsequent creation of the *TOP2B-BRAF* fusion was not detected with this array-based method. This sample was initially classified as a high-grade glioma based on a Ki-67 of 31% and very frequent mitoses however, the patient is still alive with no signs of recurrence 5 years later, which, in combination with the *BRAF* fusion, suggests that JPA is in fact the correct diagnosis.

JPA_1 was found to have a fusion between *PTPRZ1* and *BRAF* supported by 90% of barcodes on the assigned haplotype that support the SV (HAP_ALLELIC_FRAC as calculated by LongRanger, Fig. 2c). It was initially diagnosed as a high-grade glioma with a Ki-67 of 18% and very frequent mitoses. This patient also shows no signs of recurrence 7 years after the initial diagnosis and subsequent surgery. While multiple possible breakpoints were found by RNA-Seq (Additional file 2), the linked-reads supported a break in intron 15 of *PTPRZ1* and resulting in the loss of its transmembrane region and the C-terminal cytoplasmic region that contains two tyrosine-protein phosphatase domains (Fig. 1).

Pilocytic astrocytomas (PAs) are typically characterized as having quiet genomes, both in terms of somatic point mutations [42] and structural variants [6]. However, non-random aneuploidy in PAs has been described in older patients (> 14 years old), with the amplification of chromosomes 5, 7, 6, and 11 being the most common [43]. Although whole-chromosomal amplifications were not detected in samples with the canonical fusion (3–14 years old), they were found in both samples with novel *BRAF* fusion partners (Fig. 2d-e). Chromosomes 6, 7 and 10 were amplified in JPA_3 (17-year-old male) and chromosomes 6, 7, 8, 10, 11, 18 and 20 in sample JPA_1 (5-year-old female) (Fig. 2d-e). Additionally, JPA_1 was found to have 156 unique somatic events that were detected and manually validated on chromosome 7 in addition to the *PTPRZ1-BRAF* fusion (Fig. 2e). The number of structural variants detected on chromosome 7 suggests a catastrophic genomic event; however, the sample lacks the characteristic features of chromothripsis including the clustering of breakpoints and the oscillation copy number states (Fig. 2e).

Interestingly, the catastrophic event on chromosome 7 in JPA_1 was also found to contain SVs between known *BRAF* fusions partners. These include one between *KIAA1549* and* STRIP2*, were *KIAA1549* is the canonical fusion partner, one between *KIAA1549* and *SVOPL*, were *SVOPL* was found fused to *BRAF* in a case of metastatic uterus endometrial carcinoma [5], and one between *TRIM24* and *PLXNA4*, were *TRIM24-BRAF* fusions were found in two cases of metastatic colorectal cancer, one primary case of melanoma and of lung carcinoma [5]. The *TRIM24-PLXNA4* fusion was also detected by Arriba, suggesting that it is expressed in the tumor.

### Accessibility of fusion-partner genes and nuclear topology using Hi-C

**Fig. 3:**
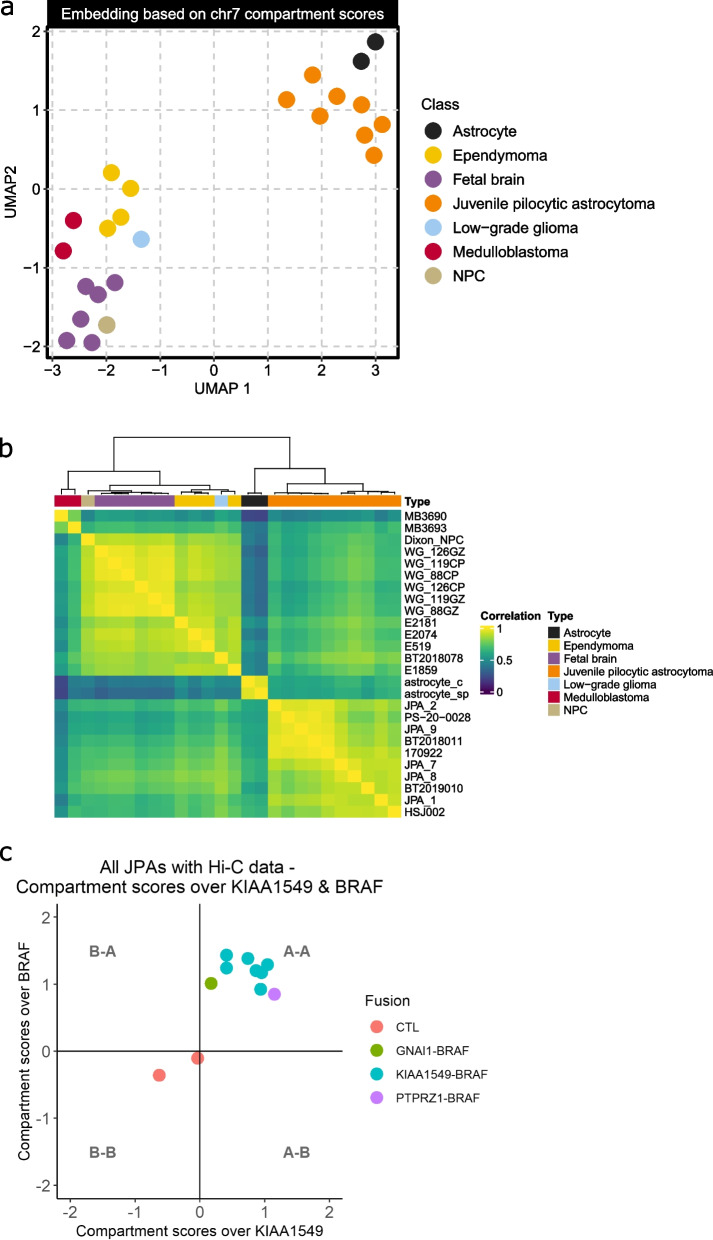
(**a**) UMAP clustering of JPAs based on the compartment scores along chromosome 7 (50 kb bins) shows two clusters separating JPAs and normal adult astrocytes from other tumor types and normal controls for other brain regions. **b** Correlation matrix showing hierarchical clustering by compartment score along chromosome 7 (50 kb bins) shows two clusters separating JPAs and normal astrocytes from other tumor types and normal controls for other brain regions. **c** Compartment score over *KIAA1549* and *BRAF* showing that both genes are in open chromatin regions in JPAs (regardless of fusion) but not in normal adult astrocytes

In order to test how chromosome compaction influences the spectrum of *BRAF* fusion partners seen in JPAs, we generated in situ Hi-C for 8 primary JPA tumors (two with linked-read data available), one primary and relapse tumor from the same patient and one cortical pediatric low-grade glioma (Supplemental Table 1). We first used the Hi-C data to generate interaction matrices to validate the *BRAF* fusions visually by plotting the interaction frequency over chromosome 7 (Supplemental Fig. 2). We also used the Hi-C data to derive the A/B compartment annotations for each chromosome were A compartments are transcriptionally active regions of the genome and B compartments are repressed [20]. We then compared the compartment annotations between the JPAs, in-house Hi-C data from other pediatric brain tumor subtypes (2 medulloblastomas and 4 posterior fossa ependymomas, 2 type A and 2 type B) as well as previously published normal adult and developing brain controls. UMAP clustering revealed high levels of correlation between compartment annotations for JPAs across the entire genome and even more so when only chromosome 7 was considered (Fig. 3a-b, Supplemental Fig. 3a-c). The JPAs also clustered closely with adult astrocytes of the cerebellum and spinal cord [34] showing that the chromosome conformation of JPAs is highly reproducible and closely resembles normal astrocytes (Fig. 3a-b).

To investigate whether open chromatin might play a role in the creation of *BRAF* fusions as suggested for somatic mutations and SVs [44, 45], we used the Hi-C data to look at the compartment scores for *KIAA1549* and *BRAF* across all JPAs (regardless of which fusion it contained) and astrocyte controls. This showed that these genes are in open chromatin regions in JPAs but not in normal adult astrocytes (Fig. 3c). We also used the breakpoints provided by the linked-read data to confirm that the SVs occurred between type A compartments for the two samples with matching Hi-C data (Supplemental Fig. 3d). As an additional validation, we looked in the single-cell expression atlas for the mouse cerebellum and confirmed expression of *BRAF* and its fusion partners in the proposed cell of origin of JPAs [40], which supports the genes being in open chromatin regions (Supplemental Note 2, Supplemental Fig. 4).

**Fig. 4:**
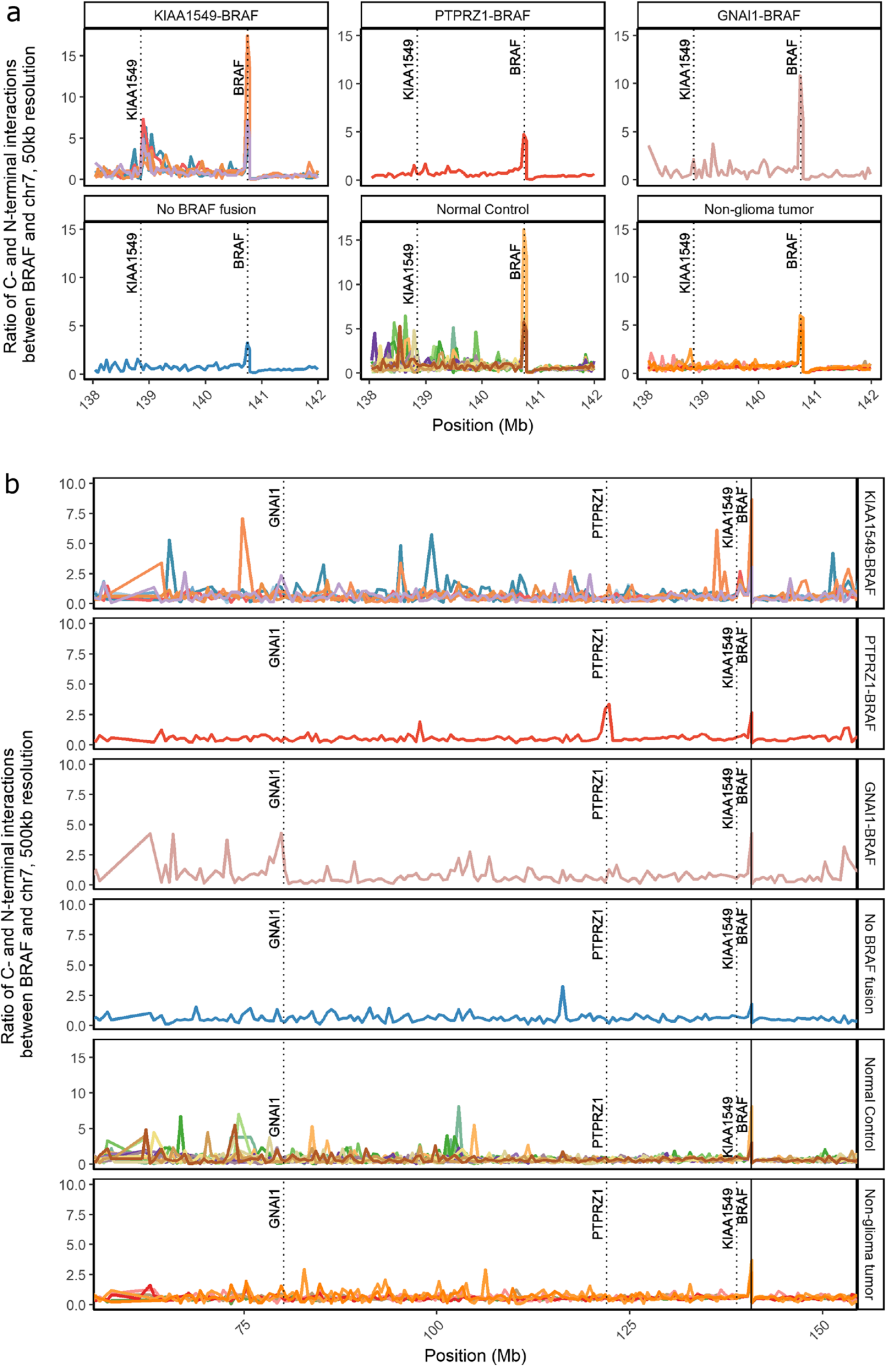
(**a**) Virtual 4C showing that the peak in the interaction between *BRAF* and *KIAA1549* is exclusive to samples with the canonical fusion (50 kb resolution). Dotted lines indicate the genomic position were a peak would represent interaction of this region with *BRAF*. **b** Virtual 4C showing that the peak in the interaction between *BRAF* and *KIAA1549* is exclusive to samples with the canonical fusion and that the samples with a novel fusion have a peak in the interaction between *BRAF* and the other fusion partner which is exclusive to those samples (500 kb resolution). Dotted lines indicate the genomic position were a peak would represent interaction of this region with *BRAF*

In order to measure the interaction of *BRAF* with regions further along chromosome 7, we generated a virtual 4C profile (see Methods). We hypothesized that since the *KIAA1549-BRAF* fusion occurs so frequently in JPA, we would see an interaction between these two regions in closely related cell types regardless of fusion statues [44]; however, this was not the case. Our analysis revealed that the interaction between *BRAF* and *KIAA1549* is exclusive to samples with the canonical fusion (Fig. 4a). Further analysis showed that in tumors with other fusion partners, the fusion could be detected by means of an exclusive interaction between *BRAF* and its fusion partner (*PTPRZ1* and *GNAI1*) in that sample (Fig. 4b).

## Discussion

In this paper, we used RNA-Seq, linked-read WGS and in situ Hi-C to characterize the genomes of 14 JPAs from 13 patients. We developed a multi-caller RNA-Seq pipeline to detect fusions which we then validated and characterized with linked-read data. We showed how high-sensitivity genomic techniques could provide further insight into the varied mechanisms that allow the creation of somatic structural variants. Additionally, we revealed that 3D genome compartmentalization is stable across JPAs and most closely resembles that of normal adult astrocyte. We also showed that fusions occur between transcriptionally active regions and that the interaction between *BRAF* and its fusion partner is exclusive to samples with an oncogenic fusion.

Our analysis also included the characterization of two tumors with novel *BRAF* fusion partners, *PTPRZ1* and *TOP2B*. *PTPRZ1* is a member of the receptor protein tyrosine phosphatase family that is highly expressed in normal brain tissue and is recurrently fused to *MET* in adult grade III astrocytomas and secondary glioblastomas [46]. In vitro work on *PTPRZ-B* knockdown cells showed that the N-terminal extracellular domain was sufficient to induce cellular migration (retained in the *PTPRZ1-BRAF* fusion) while the C-terminal is important for glioma proliferation in adults [47]. However, this was not the only structural variant found in JPA_1. While JPAs genomes are typically quiet, analysis of this tumor revealed a chromosome-shattering event. Review of the literature allowed the identification of another JPA with a highly rearranged genome; a JPA with a *FYCO1-RAF1* fusion, a *ST6GAL1-WHSC1* fusion and chromoplexy involving chromosomes 1, 3, 4, 10, 11, 12, 16 and 22 was identified by Zhang et al using a combination of RNA-Seq and WGS [6].

*TOP2B* is a topoisomerase that eliminates supercoils occurring during DNA replication and transcription by creating and repairing double-stranded breaks in the DNA [48, 49]. There is on-going work to discover anticancer drugs that could function as catalytic inhibitors for both *TOP2A* (which is essential to the cell cycle) and *TOP2B* [50]. One such example is T60 which was found to block a DNA binding pocket found in both TOP2 proteins. This DNA binding pocket is also kept in the *TOP2B-BRAF* fusion. Recent work also has shown that *TOP2B* interacts with *CTCF* and the cohesin complex, which suggests a role for *TOP2B* in chromatin organization [49]. Unfortunately, we could not study the effects of the *TOP2B-BRAF* fusion on chromatin conformation, as tumor tissue from JPA_3 was not available for Hi-C. Interchromosomal translocations account for the majority of rare oncogenic fusions detected in JPAs by WGS and/or RNA-Seq. These include *BRAF* fusions with *RNF130* [3], *CLCN6* [3], *FXR1* [6], and *MACF1* [6], *RAF1* fusions with *QKI* [6] and *NFIA* [51], and a *QKI-NTRK2* fusion [3]. However, the *TOP2B-BRAF* fusion appears to be the only one caused by the insertion of a segment from another chromosome.

To date, most genomic studies regarding JPAs have used array technologies [10–14] which do not have the sensitivity required to detect balanced or low frequency somatic events. While whole-genome and bulk RNA sequencing provide more sensitivity than arrays, extensive optimization is required in order to avoid missing low frequency events [41]. Linked-reads provide a good middle ground in the trade-off between the base calling accuracy of second-generation sequencing and the long-range information provided by third-generation sequencing. While linked-reads are more costly than RNA-Seq mainly due to increased sequencing depth, they require a fraction of the starting material (Supplemental Table 6). The linked-read protocol’s minimal input requirements (1-10 ng range) is an important consideration since JPAs requiring additional therapies are typically assessed using small size biopsies from regions outside of the cerebellum including the optic pathway and the brain midline. Linked-reads also allow the accurate detection of structural variants and point mutations, and provide haplotype information which can help detangle complex rearrangements such as in the case of the *TOP2B-BRAF* fusion. However, currently available linked-read data analysis tools need to be further developed in order to increase sensitivity and accuracy in automated workflows. It is worth noting that although the 10x Genomics linked-read technology is now discontinued, alternatives are being developed by MGI [52], Universal Sequencing [53] and others [54].

Alternatively, since the interaction between *BRAF* and its fusion partner is specific to fusion expressing samples, Hi-C could also be used as a diagnostic tool. Work is currently underway to develop a commercial chromosome conformation capture technology for formalin-fixed, paraffin-embedded (FFPE) samples with the aim of using it to detect SVs in fixed solid tumors [55]. While the ability to process fixed samples is important, for Hi-C to be of clinical use, work still needs to be done to develop tools and standard computational pipelines for automatic SV calling [55–57]. Additionally, Hi-C is currently the most expensive of the three technologies discussed in this paper so costs would need to come down to make it more accessible (Supplemental Table 6).

Some genomic studies have focused on using gene expression of single cells to characterize the subpopulations of cells found within JPAs [17, 40]. While single-cell RNA-Seq allows the consequences of *BRAF* fusions and MAPK pathway activation to be elucidated, it is not well suited to characterize the fusions themselves. Using the SMART-seq2 scRNA-seq protocol alone, which allows the sequencing of full transcripts, Reitman et al. detected a BRAF fusion in only 0–0.7% of cells although the addition of a qPCR step drastically improved the detection rate [17]. Recent methodological developments utilizing long read sequencing to address this deficiency are in development, such as scCOLOR-seq [58] and ScNaUmi-seq [59] which would provide a way to assign the expression of full-length fusion transcripts to specific cells in the near future.

## Conclusion

The development of a molecular diagnostic test able to detect the full spectrum of *BRAF* fusions quickly, with high precision and at low cost would help with the molecular diagnosis of JPAs and patient enrolment in clinical trials, when needed. Distinguishing between JPAs with the canonical and rare/novel fusions may also play an important role in treatment selection, specifically in response to one of the multiple different MEK and RAF inhibitors that are currently undergoing clinical trials for the treatment of tumors with *BRAF* rearrangements. Work done in melanoma cell lines has shown different responses to these inhibitors based on the level of *BRAF* fusion expression in the cell lines (controlled by the promoter of the 5′ partner) as well as the presence of a dimerization domain in the 5′ partner [7]. Both the linked-reads and Hi-C technologies have the potential to be useful in clinic for different reasons, the linked-reads due to the small input requirements and the Hi-C due to the ability to process FFPE samples. However, there is still important work to be done in developing high accuracy SV calling tools for these technologies before they may be of clinical relevance.

## Supplementary Information

**Additional file 1.** 

**Additional file 2.**

**Additional file 3.**

**Additional file 4.**

**Additional file 5.**

**Additional file 6.**

**Additional file 7.**

**Additional file 8.**

**Additional file 9:** **Supplemental Note 1.** Linked-reads - Technical notes

**Additional file 10:** **Supplemental Note 2.** Expression in the single-cell cerebellum cell atlas

## Data Availability

The datasets supporting the conclusions of this article are available in the European Genome-Phenome Archive (EGA) repository, under study ID EGAS00001006388. Additional Hi-C datasets for 170922, BT2018011, BT2019010, HSJ-002 and JPA_2 (also HSJ-170) can be found under study ID EGAS00001005476. Custom R scripts for the processing of fusion calls from RNA-Seq data and SV calls from linked-read data can be found here https://github.com/mzwaig/JPA_Paper

## Abbreviations

FFPE: Formalin-fixed, paraffin-embedded
HMW: High molecular weight
JPA: Juvenile pilocytic astrocytoma
MAPK: Mitogen-activated protein kinase
PA: Pilocytic astrocytomas
pLGG: Pediatric low-grade glioma
RNA-Seq: RNA sequencing
SV: Structural variants
UMI: Unique molecular identifier
WGS: Whole-genome sequencing

## References

[1] Jones DTW, Bandopadhayay P, Jabado N (2019). The Power of Human Cancer Genetics as Revealed by Low-Grade Gliomas. Annu Rev Genet.

[2] Jones DTW, Gronych J, Lichter P, Witt O, Pfister SM (2012). MAPK pathway activation in pilocytic astrocytoma. Cell Mol Life Sci.

[3] Jones DTW, Hutter B, Jäger N, Korshunov A, Kool M, Warnatz H-J (2013). Recurrent somatic alterations of FGFR1 and NTRK2 in pilocytic astrocytoma. Nat Genet.

[4] Jones DTW, Kocialkowski S, Liu L, Pearson DM, Bäcklund LM, Ichimura K (2008). Tandem duplication producing a novel oncogenic BRAF fusion gene defines the majority of pilocytic astrocytomas. Cancer Res.

[5] Ross JS, Wang K, Chmielecki J, Gay L, Johnson A, Chudnovsky J (2016). The distribution of BRAF gene fusions in solid tumors and response to targeted therapy. Int J Cancer.

[6] Zhang J, Wu G, Miller CP, Tatevossian RG, Dalton JD, Tang B (2013). Whole-genome sequencing identifies genetic alterations in pediatric low-grade gliomas. Nat Genet.

[7] Botton T, Talevich E, Mishra VK, Zhang T, Shain AH, Berquet C (2019). Genetic Heterogeneity of BRAF Fusion Kinases in Melanoma Affects Drug Responses. Cell Rep.

[8] Cin H, Meyer C, Herr R, Janzarik WG, Lambert S, Jones DT (2011). Oncogenic FAM131B-BRAF fusion resulting from 7q34 deletion comprises an alternative mechanism of MAPK pathway activation in pilocytic astrocytoma. Acta Neuropathol.

[9] Huang H, Hara A, Homma T, Yonekawa Y, Ohgaki H (2005). Altered expression of immune defense genes in pilocytic astrocytomas. J Neuropathol Exp Neurol.

[10] Sharma MK, Mansur DB, Reifenberger G, Perry A, Leonard JR, Aldape KD (2007). Distinct Genetic Signatures among Pilocytic Astrocytomas Relate to Their Brain Region Origin. Cancer Res.

[11] Lambert SR, Witt H, Hovestadt V, Zucknick M, Kool M, Pearson DM (2013). Differential expression and methylation of brain developmental genes define location-specific subsets of pilocytic astrocytoma. Acta Neuropathol.

[12] Zakrzewski K, Jarząb M, Pfeifer A, Oczko-Wojciechowska M, Jarząb B, Liberski PP (2015). Transcriptional profiles of pilocytic astrocytoma are related to their three different locations, but not to radiological tumor features. BMC Cancer.

[13] Antonelli M, Fadda A, Loi E, Moi L, Zavattari C, Sulas P (2018). Integrated DNA methylation analysis identifies topographical and tumoral biomarkers in pilocytic astrocytomas. Oncotarget..

[14] Sexton-Oates A, Dodgshun A, Hovestadt V, Jones DTW, Ashley DM, Sullivan M (2018). Methylation profiling of paediatric pilocytic astrocytoma reveals variants specifically associated with tumour location and predictive of recurrence. Mol Oncol.

[15] Sakamoto Y, Sereewattanawoot S, Suzuki A (2020). A new era of long-read sequencing for cancer genomics. J Hum Genet.

[16] Bergthold G, Bandopadhayay P, Hoshida Y, Ramkissoon S, Ramkissoon L, Rich B (2015). Expression profiles of 151 pediatric low-grade gliomas reveal molecular differences associated with location and histological subtype. Neuro-oncology..

[17] Reitman ZJ, Paolella BR, Bergthold G, Pelton K, Becker S, Jones R (2019). Mitogenic and progenitor gene programmes in single pilocytic astrocytoma cells. Nat Commun.

[18] Raabe EH, Lim KS, Kim JM, Meeker A, Mao X-G, Nikkhah G (2011). BRAF activation induces transformation and then senescence in human neural stem cells: a pilocytic astrocytoma model. Clin Cancer Res.

[19] Zheng GXY, Lau BT, Schnall-Levin M, Jarosz M, Bell JM, Hindson CM (2016). Haplotyping germline and cancer genomes with high-throughput linked-read sequencing. Nat Biotechnol.

[20] Lieberman-Aiden E, van Berkum NL, Williams L, Imakaev M, Ragoczy T, Telling A (2009). Comprehensive Mapping of Long-Range Interactions Reveals Folding Principles of the Human Genome. Science..

[21] Dobin A, Davis CA, Schlesinger F, Drenkow J, Zaleski C, Jha S (2013). STAR: ultrafast universal RNA-seq aligner. Bioinformatics..

[22] Haas BJ, Dobin A, Li B, Stransky N, Pochet N, Regev A (2019). Accuracy assessment of fusion transcript detection via read-mapping and de novo fusion transcript assembly-based methods. Genome Biol.

[23] Uhrig S, Ellermann J, Walther T, Burkhardt P, Fröhlich M, Hutter B (2021). Accurate and efficient detection of gene fusions from RNA sequencing data. Genome Res.

[24] COSMIC; Complete Fusion Export: Wellcome Trust Sanger Institute; 2004 [Available from: https://cancer.sanger.ac.uk/cosmic/download.

[25] Okonechnikov K, Imai-Matsushima A, Paul L, Seitz A, Meyer TF, Garcia-Alcalde F (2016). InFusion: Advancing Discovery of Fusion Genes and Chimeric Transcripts from Deep RNA-Sequencing Data. PLoS One.

[26] Spies N, Weng Z, Bishara A, McDaniel J, Catoe D, Zook JM (2017). Genome-wide reconstruction of complex structural variants using read clouds. Nat Methods.

[27] Elyanow R, Wu H-T, Raphael BJ (2017). Identifying structural variants using linked-read sequencing data. Bioinform (Oxford, England).

[28] Fang L, Kao C, Gonzalez MV, Mafra FA, Pellegrino da Silva R, Li M, et al. LinkedSV for detection of mosaic structural variants from linked-read exome and genome sequencing data. Nature. Communications. 2019;10(1):5585. 10.1038/s41467-019-13397-7 PMC6898185 31811119

[29] Wala JA, Bandopadhayay P, Greenwald NF, O'Rourke R, Sharpe T, Stewart C (2018). SvABA: genome-wide detection of structural variants and indels by local assembly. Genome Res.

[30] Viswanathan SR, Ha G, Hoff AM, Wala JA, Carrot-Zhang J, Whelan CW (2018). Structural Alterations Driving Castration-Resistant Prostate Cancer Revealed by Linked-Read Genome Sequencing. Cell..

[31] Rao Suhas SP, Huntley Miriam H, Durand Neva C, Stamenova Elena K, Bochkov Ivan D, Robinson James T (2014). A 3D Map of the Human Genome at Kilobase Resolution Reveals Principles of Chromatin Looping. Cell..

[32] Rajarajan P, Borrman T, Liao W, Schrode N, Flaherty E, Casiño C (2018). Neuron-specific signatures in the chromosomal connectome associated with schizophrenia risk. Science..

[33] The ENCODE Project Consortium (2012). An integrated encyclopedia of DNA elements in the human genome. Nature..

[34] Experiment summary for ENCSR011GNI, HiC experiment done on astrocyte of the cerebellum [Internet]. ENCODE. 2017. Available from: https://www.encodeproject.org/experiments/ENCSR011GNI/. Cited June 27, 2022

[35] Durand NC, Shamim MS, Machol I, Rao SSP, Huntley MH, Lander ES (2016). Juicer Provides a One-Click System for Analyzing Loop-Resolution Hi-C Experiments. Cell Syst.

[36] Hi-C LS, Formats D, Bicciato S, Ferrari F (2022). Hi-C Data Analysis: Methods and Protocols.

[37] Vitzthum C, Abdennur N, Lee S, Kerpedjiev P. hic2cool. 2017. https://github.com/4dn-dcic/hic2cool

[38] Venev S, Abdennur N, Goloborodko A, Flyamer I, Fudenberg G, Nuebler J, et al. open2c/cooltools: v0.5.0rc2 (v0.5.0rc2). 2021. https://github.com/qenvio/dryhic

[39] Vidal E, le Dily F, Quilez J, Stadhouders R, Cuartero Y, Graf T (2018). OneD: increasing reproducibility of Hi-C samples with abnormal karyotypes. Nucleic Acids Res.

[40] Vladoiu MC, El-Hamamy I, Donovan LK, Farooq H, Holgado BL, Sundaravadanam Y, et al. Childhood cerebellar tumours mirror conserved fetal transcriptional programs. Nature. 2019. 10.1038/s41586-019-1158-7 PMC6675628 31043743

[41] Sommerkamp AC, Uhrig S, Stichel D, St-Onge P, Sun P, Jäger N, et al. An optimized workflow to improve reliability of detection of KIAA1549:BRAF fusions from RNA sequencing data. Acta Neuropathol. 2020. 10.1007/s00401-020-02167-1 PMC7360662 32476062

[42] Alexandrov LB, Nik-Zainal S, Wedge DC, Aparicio SAJR, Behjati S, Biankin AV (2013). Signatures of mutational processes in human cancer. Nature..

[43] Fontebasso AM, Shirinian M, Khuong-Quang D-A, Bechet D, Gayden T, Kool M (2015). Non-random aneuploidy specifies subgroups of pilocytic astrocytoma and correlates with older age. Oncotarget..

[44] Grzeda KR, Royer-Bertrand B, Inaki K, Kim H, Hillmer AM, Liu ET (2014). Functional chromatin features are associated with structural mutations in cancer. BMC Genomics.

[45] Tiong K-L, Yeang C-H (2018). Explaining cancer type specific mutations with transcriptomic and epigenomic features in normal tissues. Sci Rep.

[46] Bao ZS, Chen HM, Yang MY, Zhang CB, Yu K, Ye WL (2014). RNA-seq of 272 gliomas revealed a novel, recurrent PTPRZ1-MET fusion transcript in secondary glioblastomas. Genome Res.

[47] Bourgonje AM, Navis AC, Schepens JT, Verrijp K, Hovestad L, Hilhorst R (2014). Intracellular and extracellular domains of protein tyrosine phosphatase PTPRZ-B differentially regulate glioma cell growth and motility. Oncotarget..

[48] Linka RM, Porter AC, Volkov A, Mielke C, Boege F, Christensen MO (2007). C-terminal regions of topoisomerase IIalpha and IIbeta determine isoform-specific functioning of the enzymes in vivo. Nucleic Acids Res.

[49] Uusküla-Reimand L, Hou H, Samavarchi-Tehrani P, Rudan MV, Liang M, Medina-Rivera A (2016). Topoisomerase II beta interacts with cohesin and CTCF at topological domain borders. Genome Biol.

[50] Matias-Barrios VM, Radaeva M, Song Y, Alperstein Z, Lee AR, Schmitt V, et al. Discovery of New Catalytic Topoisomerase II Inhibitors for Anticancer Therapeutics. Front. Oncol. 2021:10. 10.3389/fonc.2020.633142 PMC7883873 33598437

[51] Rivera B, Gayden T, Carrot-Zhang J, Nadaf J, Boshari T, Faury D (2016). Germline and somatic FGFR1 abnormalities in dysembryoplastic neuroepithelial tumors. Acta Neuropathol.

[52] Wang O, Chin R, Cheng X, Wu MKY, Mao Q, Tang J (2019). Efficient and unique cobarcoding of second-generation sequencing reads from long DNA molecules enabling cost-effective and accurate sequencing, haplotyping, and de novo assembly. Genome Res.

[53] Chen Z, Pham L, Wu TC, Mo G, Xia Y, Chang PL (2020). Ultralow-input single-tube linked-read library method enables short-read second-generation sequencing systems to routinely generate highly accurate and economical long-range sequencing information. Genome Res.

[54] Redin D, Frick T, Aghelpasand H, Käller M, Borgström E, Olsen R-A (2019). High throughput barcoding method for genome-scale phasing. Sci Rep.

[55] Troll CJ, Putnam NH, Hartley PD, Rice B, Blanchette M, Siddiqui S (2019). Structural Variation Detection by Proximity Ligation from Formalin-Fixed. Paraffin-Embedded Tumor Tissue J Mol Diagn.

[56] Dixon JR, Xu J, Dileep V, Zhan Y, Song F, Le VT (2018). Integrative detection and analysis of structural variation in cancer genomes. Nat Genet.

[57] Mallard C, Johnston MJ, Bobyn A, Nikolic A, Argiropoulos B, Chan JA, et al. Hi-C detects genomic structural variants in peripheral blood of pediatric leukemia patients. Cold Spring Harbor molecular case studies. 2022;8(1). 10.1101/mcs.a006157 PMC8744495 34819303

[58] Philpott M, Watson J, Thakurta A, Brown T, Brown T, Oppermann U (2021). Nanopore sequencing of single-cell transcriptomes with scCOLOR-seq. Nat Biotechnol.

[59] Lebrigand K, Magnone V, Barbry P, Waldmann R (2020). High throughput error corrected Nanopore single cell transcriptome sequencing. Nat Commun.

