# Supplementary material for: Detection and genomic analysis of BRAF fusions in Juvenile Pilocytic Astrocytoma through the combination and integration of multi-omic data
Source: BMC Cancer. 2022 Dec 12;22:1297. doi: 10.1186/s12885-022-10359-z (PMC9743522; doi:10.1186/s12885-022-10359-z)
Supplement: Supplementary file 1 — Additional file 1. [file 12885_2022_10359_MOESM1_ESM.pdf]

Detection and genomic analysis of BRAF fusions in Juvenile Pilocytic Astrocytoma through the combination and integration of multi-omic data - Supplemental Figures (1-4) and Tables (1-6)

Supplemental Figure legends

Sup Fig 1 Linked-reads data supporting *BRAF* fusions, visualized in Loupe, of tandem duplication resulting in the canonical *KIAA1549-BRAF* fusion in JPA_4, JPA_5, JPA_6

Sup Fig 2 Interaction matrices showing interactions between (**a**) *KIAA1549* and *BRAF* in fused and non-fused JPAs, (**b**) *PTPRZ1* and *BRAF* in JPA_1 and (**c**) *GNAI1* and *BRAF* in JPA_9

Sup Fig 3 (**a**) UMAP clustering of JPAs based on the compartment scores for the entire genome (50kb bins) shows clustering of JPAs. (**b**) Correlation matrix showing hierarchical clustering by compartment score over entire genome (50kb bins) shows clustering of JPAs. (**c**) Outer, Compartment scores for adult astrocytes and JPAs across chromosomes 3 and 7. Inner, most common *RAF* fusions in JPAs as well as novel fusions described in this paper. (**d**) Compartment score over breakpoints in *KIAA1549* and *BRAF* in JPA_2, and in *PTPRZ1* and *BRAF* in JPA_1 showing that both genes are in open chromatin regions in the matched Hi-C data

Sup Fig 4 (**a**) Expression of *BRAF* across all developmental time points regardless of cell type. (**b**) Average expression of *BRAF* across all cell types in the mouse cerebellum single-cell atlas at time points E16 ad E18. (**c**) Percent cells expressing *BRAF* across all cell types in the mouse cerebellum single-cell atlas at time points E16 ad E18. (**d**) Expression of *BRAF*, *RAF1*, recurrent and novel fusion partners in all cell types in the mouse cerebellum single-cell atlas at time points E16 and E18

Supplemental Figure 1.

JPA_4
Tandem duplication resulting in
KIAA1549-BRAF
(EX16-EX9,HAP_ALLELIC_FRAC=1.0)

JPA_5
Tandem duplication resulting in
KIAA1549-BRAF
(EX16-EX9, HAP_ALLELIC_FRAC=0.928)

JPA_6
Tandem duplication resulting in
KIAA1549-BRAF
(EX16-EX9, HAP_ALLELIC_FRAC=0.8)

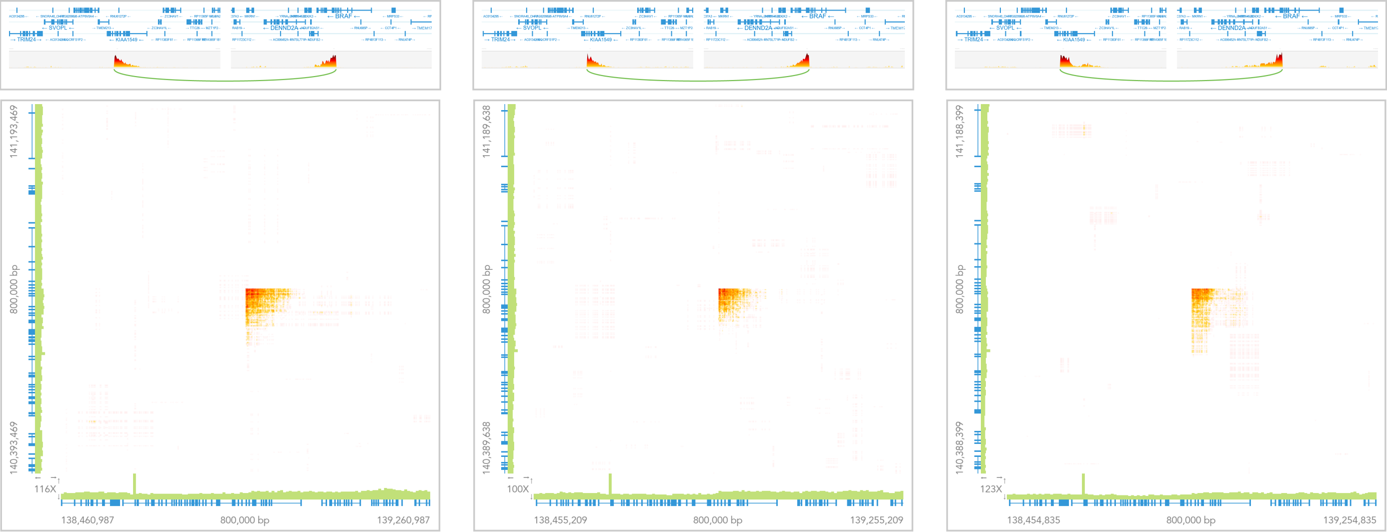

Supplemental Figure 2.

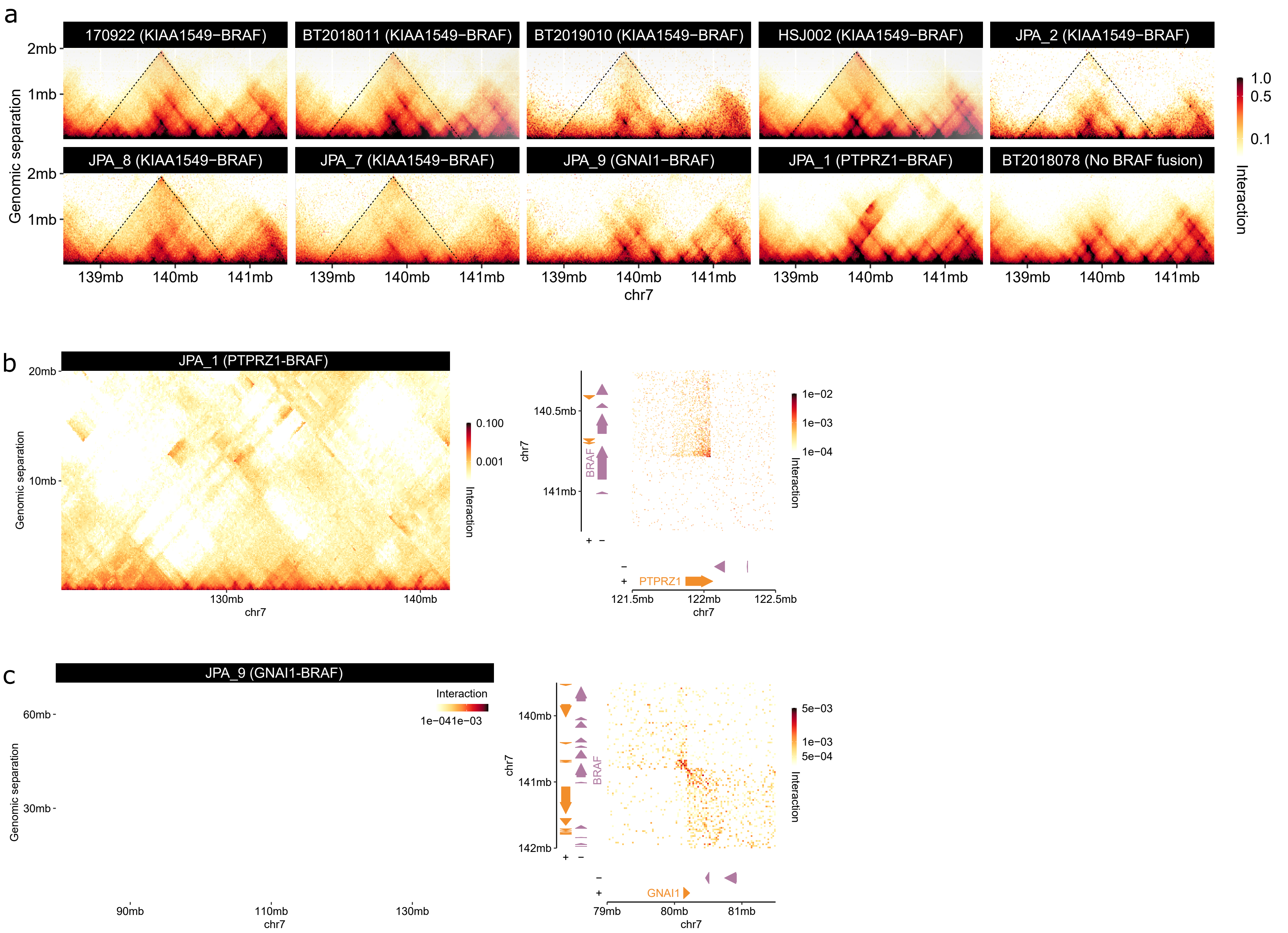

Supplemental Figure 3.

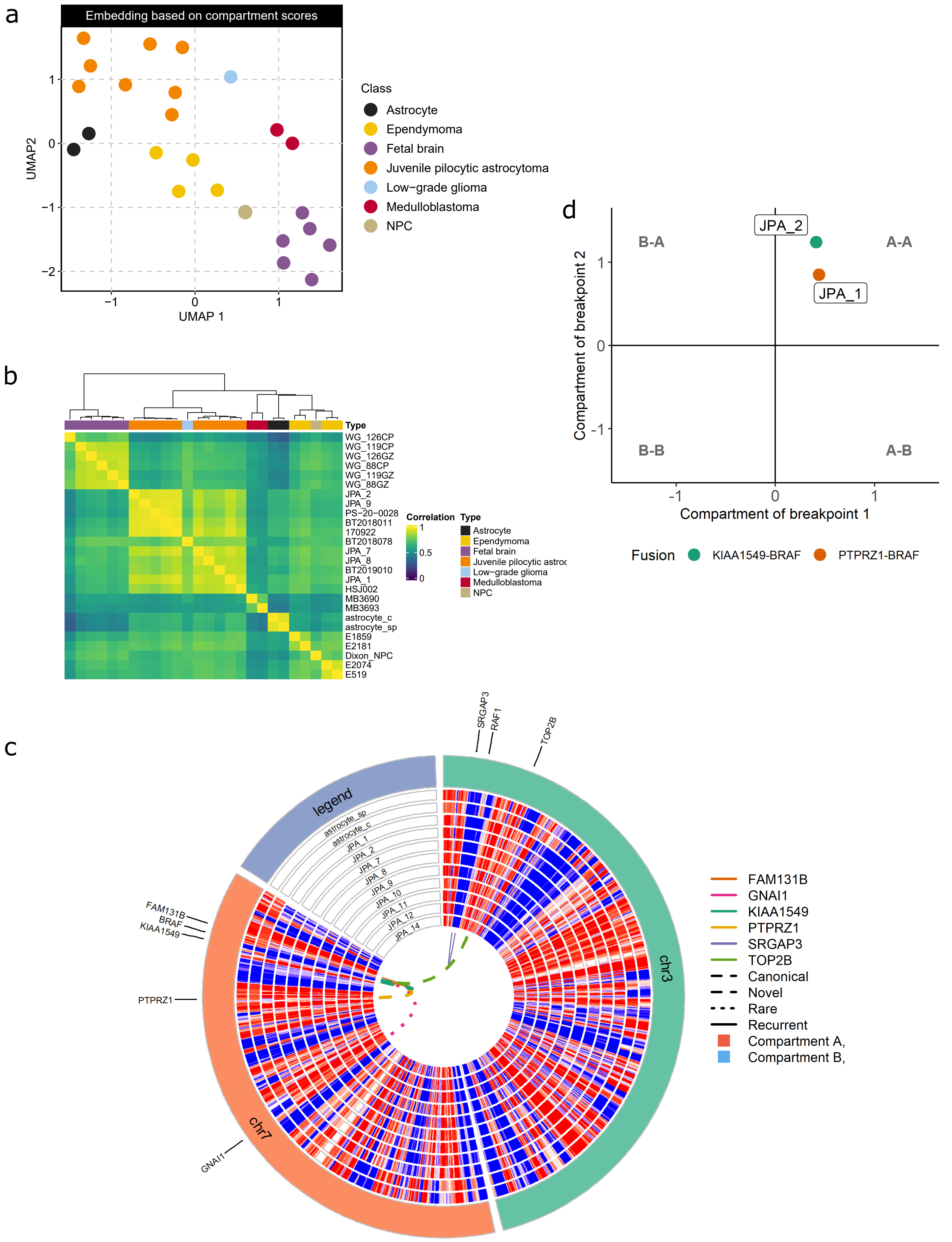

Supplemental Figure 4.

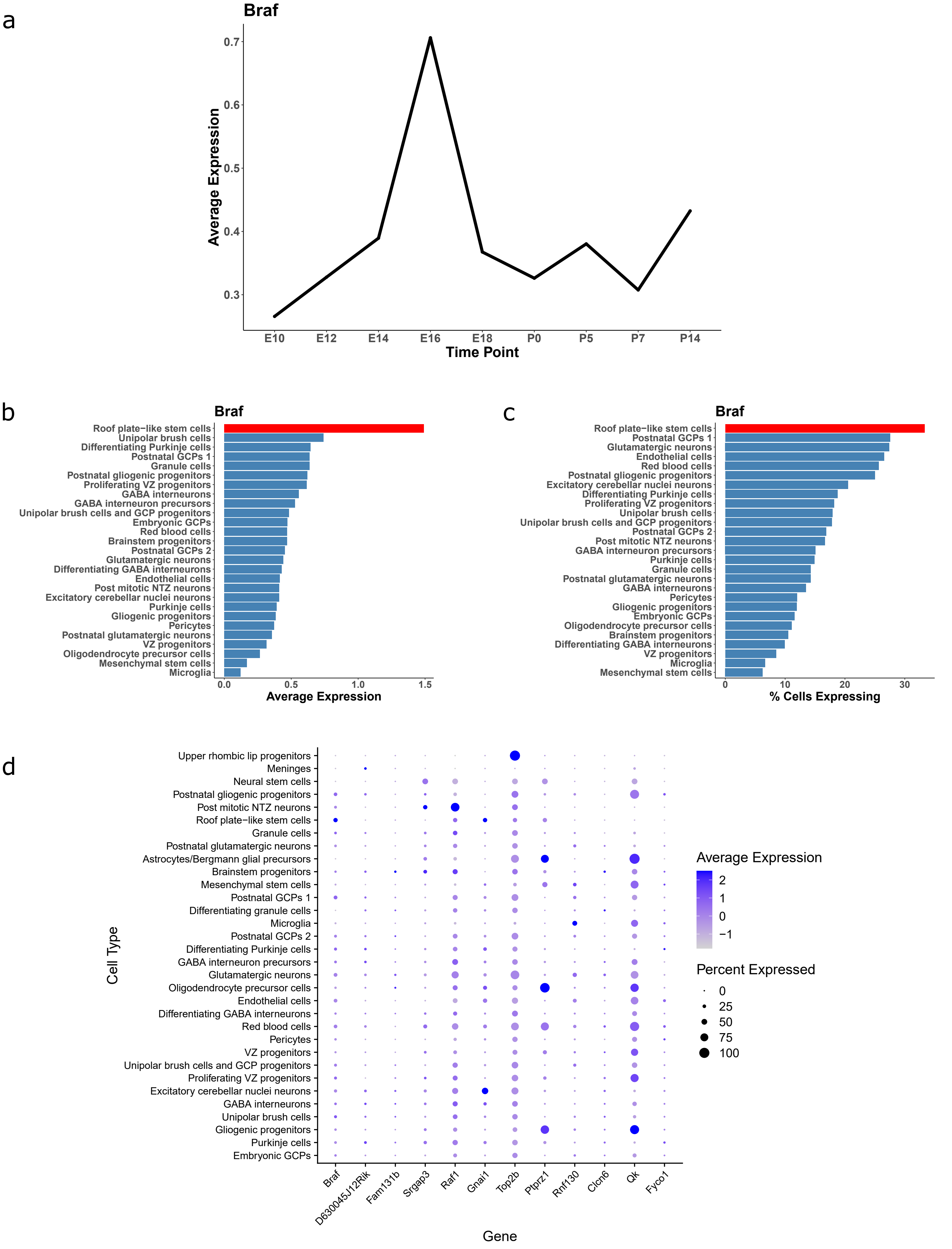

Supplemental Table 1. Sample description and detection of BRAF fusions across technologies

Sample Information				Linked-reads					RNA-Seq			Hi-C
**Patient**	**Diagnosis**	**Tumor Location**	**5' Fusion Partner**	**LongRanger**	**GROC_SV**	**LinkedSV**	**NAIBR**	**SvABA**	**STAR-Fusion**	**Arriba**	**InFusion**	**Hi-C**
**JPA_1**	JPA	Cerebellum	PTPRZ1	Yes	No	No	Yes	No	Yes	Yes	Yes	Yes
**JPA_2**	JPA	Left cerebellum	KIAA1549	Yes	No	No	Yes	Yes	No	Yes	Yes	Yes
**JPA_3**	JPA	Posterior	TOP2B	Yes	Yes	No	Yes	Yes	Yes	Yes	No	-
**JPA_4**	JPA	Thalamic Midbrain	KIAA1549	Yes	Yes	No	Yes	Yes	No	Yes	No	-
**JPA_5**	JPA	Posterior fossa	KIAA1549	Yes	Yes	No	Yes	Yes	No	Yes	No	-
**JPA_6**	JPA	Posterior fossa	KIAA1549	Yes	Yes	No	Yes	No	No	Yes	No	-
**JPA_7**	JPA	Ventricle	KIAA1549	-	-	-	-	-	No	Yes	No	Yes
**JPA_8**	Recurrence of JPA_7		KIAA1549	-	-	-	-	-	-	-	-	Yes
**JPA_9**	JPA	Spinal	GNAI1	-	-	-	-	-	Yes	Yes	No	Yes
**HSJ-002**	JPA	Cerebellum	KIAA1549	-	-	-	-	-	No	Yes	No	Yes
**BT2018011**	JPA	Posterior fossa	KIAA1549	-	-	-	-	-	-	-	-	Yes
**170922**	JPA	Trigone	KIAA1549	-	-	-	-	-	-	-	-	Yes
**BT2019010**	JPA	Posterior fossa	KIAA1549	-	-	-	-	-	-	-	-	Yes
**BT2018078**	LGG	Cortex	No Fusion	-	-	-	-	-	-	-	-	Yes

Supplemental Table 2. Fusion calls in RNA-Seq data

Sample	Star-Fusion	Arriba	InFusion	Total calls
**JPA_1**	10	851	107	968
**JPA_2**	1	859	45	905
**JPA_3**	1	505	28	534
**JPA_4**	0	779	157	936
**JPA_5**	1	1103	81	1185
**JPA_6**	1	1124	171	1296
**JPA_7**	1	515	34	550
**JPA_9**	1	635	41	677
**HSJ-002**	1	404	24	429

Supplemental Table 3. SV calls in linked-read data

Sample	Tissue	GROC_SV	LinkedSV	LongRanger	NAIBR	SvABA
**JPA_1**	Blood	41	148	215	3313	401366
**JPA_1**	Tumor	40	157	1290	9605	9947
**JPA_2**	Blood	15	122	144	5198	59193
**JPA_2**	Tumor	10	228	957	5668	1971
**JPA_3**	Blood	31	96	254	6764	40187
**JPA_3**	Tumor	4	47	691	2669	2403
**JPA_4**	Blood	15	92	144	7778	30027
**JPA_4**	Tumor	3	141	382	4061	3514
**JPA_5**	Blood	22	103	164	4708	31948
**JPA_5**	Tumor	3	99	706	3155	556
**JPA_6**	Blood	22	71	145	4684	36062
**JPA_6**	Tumor	11	334	1211	3068	3320

Supplemental Table 4. Extraction method and sample metrics as calculated by LongRanger

	Tumor								Blood							
**Sample**	**Extraction & Size selection method**	**Mean depth**	**Average molecule length**	**DNA in Molecules >20kb**	**DNA in Molecules >100kb**	**SNPs Phased**	**N50 Phase Block**	**Number of large SVs**	**Extraction & Size selection method**	**Mean depth**	**Average molecule length**	**DNA in Molecules >20kb**	**DNA in Molecules >100kb**	**SNPs Phased**	**N50 Phase Block**	**Number of large SVs**
**JPA_1**	Chemagen & BluePippin PacBio 20kb	77.1X	19kb	49.7%	3.03%	99.5%	460kb	1378	MagAttract & BluePippin BPLUS10	69.7X	42kb	86.9%	3.94%	99.60%	1,946kb	186
**JPA_2**	MagAttract	38.7X	58kb	90.20%	16.7%	98.90%	2,981kb	986	MagAttract	32.7X	96kb	95.00%	49.1%	99.00%	8,996kb	151
**JPA_3**	Chemagen & BluePippin PacBio 20kb	38.8X	31kb	78.90%	1.75%	99.0%	816kb	661	MagAttract & HLS HMW HighPass 300kb	31.4X	79kb	94.50%	33.5%	98.9%	5,580kb	265
**JPA_4**	MagAttract	33.1X	85kb	95.30%	39.40%	99.4%	5,874kb	398	MagAttract	29.4X	104kb	96.20%	55.2%	99.3%	8,018kb	141
**JPA_5**	MagAttract	27.6X	64kb	93.30%	22.00%	98.90%	3,067kb	681	MagAttract	35.1X	84kb	94.90%	38.6%	99.00%	5,828kb	141
**JPA_6**	MagAttract	36.8X	62kb	89.20%	22.30%	99.30%	3,888kb	1273	MagAttract	30.4X	76kb	94.10%	33.2%	99.3%	4,605kb	121

Supplemental Table 5. Quality metrics for Hi-C datasets

**Sample**	**Resolution**	**total_sequenced_abs**	**normal_paired_abs**	**normal_paired_pct**	**chimeric_paired_abs**	**chimeric_paired_pct**	**alignable_abs**	**alignable_pct**	**hic_contacts_abs**	**hic_contacts_pct**	**ligation_motif_pct**	**inter_chrom_abs**	**inter_chrom_pct**	**intra_chrom_abs**	**intra_chrom_pct**	**short_range_abs**	**short_range_pct**	**long_range_abs**	**long_range_pct**
**JPA_1**	4900	648298450	434151379	66.97	172596578	26.62	606747957	93.59	413876697	71.79	34.47	90133632	15.63	323743065	56.16	120844319	20.96	202898561	35.2
**JPA_2**	17000	521941348	381214913	73.04	108938726	20.87	490153639	93.91	117676846	48.43	15.58	21018373	8.65	96658473	39.78	71942152	29.61	24716234	10.17
**JPA_7**	14800	299896108	136896650	45.65	107227035	35.75	244123685	81.4	176270792	74.7	55.68	72731084	30.82	103539708	43.88	39900390	16.91	63639169	26.97
**JPA_8**	12200	291618237	152941765	52.45	85616955	29.36	238558720	81.81	147045245	67.8	40.56	45537216	21	101508029	46.8	37328144	17.21	64179749	29.59
**JPA_9**	16850	351523017	210792802	59.97	87226064	24.81	298018866	84.78	148145954	51.99	25.15	55833407	19.59	92312547	32.39	44334593	15.56	47977733	16.84
**HSJ002**	4850	759595762	562263832	74.02	138897082	18.29	701160914	92.31	402037505	61.13	18.18	112588791	17.12	289448714	44.01	97622344	14.84	191826080	29.17
**BT2018011**	5550	487812373	245369596	50.3	201712579	41.35	447082175	91.65	319948048	83.02	55.38	101523797	26.34	218424251	56.67	50983489	13.23	167440693	43.45
**170922**	5900	510715657	247098337	48.38	215059964	42.11	462158301	90.49	315888184	83.34	57.15	108013239	28.5	207874945	54.84	61782464	16.3	146092344	38.54
**BT2019010**	42500	54872613	27603607	50.3	23221998	42.32	50825605	92.62	36439117	83	56.82	11154120	25.41	25284997	57.59	6460655	14.72	18824330	42.88
**BT2018078**	7600	365683475	178617177	48.84	154954061	42.37	333571238	91.22	244592496	83.59	58.06	69628651	23.8	174963845	59.79	46626070	15.93	128337700	43.86

Supplemental Table 6. Comparison of RNA-Seq, 10x Genomics Linked-Reads and Hi-C protocols

Technology	Preparation of input material	Amount of starting material	Hands on time for preparation of input material	Time for library preparation	Total turnaround time (not including sequencing)	Reagent Cost per library	Input Requirements	Recommended Sequencing depth	Recommended Sequencing Length
**RNA-Seq**	RNA extraction with RNeasy Mini Kit (1h)<br>QC by Qubit and TapeStation (1h)	10–30mg	2 hours	~10.5 hours	2 days	$	100-1000ng total RNA	50-100 million paired reads	75-150bp
**10X linked-Reads**	HMW DNA extraction with circulomics kit (2h)<br>Recommended to allow HMW DNA to sit and resuspend overnight<br>QC by Qubit and Femto Pulse (1h prep, 3h run time)	5-10mg	3 hours<br>(overnight incubation for resuspension is recommended)	8 hours	3 days	$$	10ng HMW DNA	425 million paired reads	150bp
**Hi-C from fresh/frozen tissue**	Dovetail® Omni-C Kit (Steps 1-3)<br>Tissue dissociation with liquid Nitrogen and Crosslinking (2h)<br>Lysate QC (2h)<br>Proximity Ligation (4.5h)	20mg	9.5 hours	5 hours	2 days	$$$	150ng purified DNA	300 million paired reads	150bp
**Hi-C from FFPE**	Arima-HiC+ FFPE Kit<br>Dewaxing (1h)<br>Digestion (3h)<br>Overnight incubation<br>Reverse Cross-Linking (15min)<br>Overnight incubation<br>Purification and QC (1h)	$5mm^3$ from 5-10µm FFPE tissue section	5.5 hours	4 hours	3 days	$$$	125ng-2000ng purified, frqagmented and size selected DNA	600 million paired reads	150bp
