# Supplementary material for: Detection and genomic analysis of BRAF fusions in Juvenile Pilocytic Astrocytoma through the combination and integration of multi-omic data
Source: BMC Cancer. 2022 Dec 12;22:1297. doi: 10.1186/s12885-022-10359-z (PMC9743522; doi:10.1186/s12885-022-10359-z)
Supplement: Supplementary file 9 — Additional file 9: Supplemental Note 1. Linked-reads - Technical notes [file 12885_2022_10359_MOESM9_ESM.docx]

Supplemental Note 1: Linked-reads - Technical notes

Maximizing the information that can be acquired from a linked-read library requires using the largest fragments of DNA possible. We mainly utilized Qiagen’s MagAttract HMW DNA kit (>150kb), recommended by 10x Genomics, for tissue and blood extractions. However, preforming the extraction in house is not always possible and, as our samples are clinical samples, they may not always be of high quality. To improve the quality of the HMW DNA, subpar samples were size selected using either the BluePippin PacBio 20kb cassette or SageHLS HighPass >300kb protocol (Supplemental Table 2). Since the generation of these samples, we have mainly switched to using the Circulomics Nanobind Tissue Big DNA Kit (Circulomics Inc, Baltimore, Maryland, United States, cat# SKU NB-900-701-01) followed by their Short Read Eliminator Kit (cat# SKU SS-100-101-01) for our in-house DNA extractions which provides both high yield and very long DNA fragments.

Initially, the linked-read data was aligned to genome build 37 with the LongRanger pipeline which made between 398-1378 large SV calls (>30kb) per tumor and 121-265 large SV calls per matching blood sample. However, only 3/6 *BRAF* fusions causing SVs were called by LongRanger despite all being visually validated in Loupe. This lead to the decision to create a multi-caller pipeline to improve sensitivity. After running our custom pipeline consisting of SV callers LongRanger, GROC-SV ([26](#_ENREF_26)), NAIBR ([27](#_ENREF_27)), LinkedSV ([28](#_ENREF_28)) and SvABA ([29](#_ENREF_29)), 5/6 *BRAF* fusions causing SVs were called by at least 2 callers out of 1996-5308 large SV calls per tumor (>10kb). Once we updated to genome build 38, all 6 SVs were called by LongRanger, however, 382-1290 large SV calls were still made per tumor (Supplemental Table 3). When we ran our multi-caller pipeline, all SVs were called by at least 2 callers and 5/6 were called by at least 3 callers highlighting the need for an automated multi-caller approach to 10X linked-read data (Supplemental Table 1, 3, Additional files 3-8).
