# Supplementary material for: Detection and genomic analysis of BRAF fusions in Juvenile Pilocytic Astrocytoma through the combination and integration of multi-omic data
Source: BMC Cancer. 2022 Dec 12;22:1297. doi: 10.1186/s12885-022-10359-z (PMC9743522; doi:10.1186/s12885-022-10359-z)
Supplement: Supplementary file 10 — Additional file 10: Supplemental Note 2. Expression in the single-cell cerebellum cell atlas [file 12885_2022_10359_MOESM10_ESM.docx]

Supplemental Note 2: Expression in the single-cell cerebellum cell atlas

To further confirm that both *BRAF* and its various fusion partners occur in open chromatin regions simultaneously, we asked whether they are co-expressed in a cell type and developmental stage specific manner. We used the single-cell expression atlas for the mouse cerebellum from Vladoiu *et al*. ([40](#_ENREF_40)) to look at the expression of *BRAF* and its fusion partners across all time points for all cell types since there is evidence that the chances of creating a somatic mutation are highest when genes are being actively transcribed and in open chromatin ([44](#_ENREF_44), [45](#_ENREF_45)). Despite the dropout rate of single-cell transcriptomic datasets and the challenges when investigating the expression of individual genes, *BRAF* was detected in roof plate-like stem cells at E16 and E18, the postulated cell-of-origin ([40](#_ENREF_40)), where it displayed the highest expression compared to other time points and cell types (Supplemental Figure 4a-b). Furthermore, at E16 and E18, the glial cell lineage, which includes roof plate-like stem cells, proliferating VZ progenitors and gliogenic progenitors, also shows expression of recurrent and novel mouse homologues of *BRAF* fusion partners such as *FAM131B*, *SRGAP3, GNAI1, RNF130, CLCN6, QKI, FYCO1, TOP2B*, and *PTPRZ1,* suggesting that these genes are also in open chromatin regions (Supplemental Figure 4c-d). We propose that, if *BRAF* expression peaks at E16 then decreases, it follows that this is when you have the highest chances of a break occurring in *BRAF*. This break will then be repaired with a second nearby open chromatin region and (depending on the region used for repair) a *BRAF* fusion can be formed. Since the BRAF fusion is regulated by the promoter of its fusion partner, the expression level of the fusion partner will then dictate if the cell propagates to form a tumor or cell senescence because of overexpression of catalytically active *BRAF* ([18](#_ENREF_18)).
